# The effects of early combined training on the physical development of preterm infants with different gestational ages

**DOI:** 10.3389/fped.2023.1066751

**Published:** 2023-03-03

**Authors:** Fang He, Na Wu, Xiuwei Ma, Xiaofang Liu, Ming Gao, Zhichun Feng

**Affiliations:** ^1^ Faculty of Pediatrics, The Chinese PLA General Hospital, Beijing, China; ^2^ Department of Child Development, The seventh Medical Center of PLA General Hospital, Beijing, China

**Keywords:** oral training, breathing training, soft palate lifting function training, preterm infants, physical development

## Abstract

**Objective:**

To investigate the effects of early combined training on the physical development of preterm infants of different gestational ages.

**Methods:**

A total of 144 preterm infants from our hospital's neonatal intensive care unit (NICU) between 2019 and 2020 were selected as the research participants and randomly divided into an intervention group (77 cases) and a control group (67 cases). The physical development and catch-up growth satisfaction rate of preterm infants in the intervention and control groups were compared at 40 weeks, 3 months, 6 months and 12 months of corrected age.

**Results:**

At 40 weeks of gestational age and corrected 3 months of gestational age, the physical growth indexes of the intervention group were higher than those of the control group, with a statistical difference (*P* < 0.05). At the corrected age of 12 months, the body weight and length of preterm infants in the <29 weeks intervention group were still higher than those in the control group (*P* < 0.05). The body weight of preterm infants in the 29–32 weeks and 32–34 weeks intervention groups was higher than in the control group (*P* < 0.05). There was no statistical difference between the intervention and control groups in the 34–37 weeks category (*P* > 0.05). The catch-up growth satisfaction rates of all the physical growth indexes in the intervention group were higher than those of the control group at the corrected 3 months for all the gestational ages (*P* < 0.05). While those indexes in the three >29 weeks intervention groups were higher than those in the <29 weeks intervention group at the corrected age of 12 months (*P* < 0.05).

**Conclusion:**

Early combined training can promote the early catch-up growth of preterm infants, especially in the early gestational age groups (<34 weeks), and the catch-up growth promotion can last for 12 months. The older the gestational age, the sooner catch-up growth satisfaction will occur.

## Key messages

1. **Preterm infants** are prone to sucking and swallowing dysfunction, as well as sucking–swallowing–breathing disorder, which leads to difficulty in oral feeding and/or feeding intolerance.
2. **Oral training** can promote the sucking and swallowing function of preterm infants, while **breathing training** can promote the coordination of breathing, sucking and swallowing, and **the soft palate** is involved in breathing, eating and speaking.
3. **Early combined training** is an **ultra-early intervention** that can promote the early catch-up growth of preterm infants, particularly in the small gestational age group, and the catch-up growth promotion can last until 12 months.

## 1. Introduction

According to the World Health Organization (WHO), nearly 15 million preterm babies are born each year worldwide, accounting for 10% of all newborns (1, 2). With the development of medical technology in the neonatal intensive care unit (NICU), the survival rate of preterm infants is getting higher and higher. Paying attention to the healthy growth of preterm infants has become a medical and social problem (3, 4). Due to immature nervous system development, preterm infants are prone to sucking and swallowing dysfunction, which leads to difficulty in oral feeding and/or feeding intolerance (5). Although tube feeding and parenteral nutrition can also meet the nutritional needs of preterm infants, they reduce or eliminate all or part of the digestive tract's stimulation, resulting in a variety of adverse reactions. Full oral feeding is the main index of preterm infants discharged from the hospital and the ultimate goal aim at fulfilling their nutritional needs. Oral feeding can effectively promote the maturity of the sucking and swallowing reflexes of preterm infants and promote their growth and development (6).

Studies have pointed out that oral training can promote the sucking and swallowing function of preterm infants and positively affect feeding difficulty in infants (7). Breathing training intervention mainly involves a series of regular and complete breathing training and coordination exercise between breathing and swallowing. The soft palate is involved in many of our most basic functions, such as breathing, eating and speaking. Previous studies on oral training mostly refer to the oral exercise program formulated by Fucile et al. (8), i.e., finger stimulation exercise, while breathing training and soft palate lifting function training are neglected. Zelda Greene (9) analysed 19 studies on the oral stimulation of preterm infants in 2017 and found that none paid attention to the catch-up speed and long-term physical development of preterm infants.

Accordingly, we conducted the current research study, selecting an ultra-early research intervention time when the vital signs of preterm infants were stable. We combined the traditional oral training method with breathing training and soft palate lifting function training, which we called early combined training. Each of the basic training types is a recognised protocol. Previous studies have included oral training plus breathing training, but this is the first time that the three methods are integrated. The combined training is conducted by a speech trainer who has received professional instruction. Preterm infants in the NICU of our hospital were randomly selected for early combined training and divided into groups according to gestational age. After discharge, the patients were followed up regularly until the corrected age of 12 months. The effects of early combined training on the early and long-term physical development of preterm infants, and the catch-up growth of preterm infants with different gestational ages, were observed to standardise oral intervention procedures and set standards for preterm infants in the NICU.

## 2. Materials and methods

### 2.1. General information

Convenience sampling was used to select preterm infants hospitalised in our NICU from 2019 to 2020 as the study participants, who were regularly followed up until 12 months of correction age in the child health clinic according to the follow-up time point after discharge. The participants were divided into the intervention group (*n* = 77) and the control group (*n* = 67) according to the parity test number of randomly generated numbers. The inclusion criteria were as follows: (1) preterm infant, gestational age <37 weeks; (2) Apgar score >7; (3) without oral feeding; (4) no congenital malformation or serious complications; (5) stable vital signs. The exclusion criteria were as follows: (1) patients with chromosomal abnormalities, nervous system malformations, complex congenital heart disease, congenital digestive tract malformations or other congenital diseases; (2) neurological complications, such as grade III or IV intracranial haemorrhage or periventricular leukomalacia; (3) severe infection, disseminated intravascular coagulation, circulatory failure; (4) surgical conditions caused by other serious complications, such as necrotising enterocolitis (NEC); (5) obvious respiratory diseases, such as bronchopulmonary dysplasia and malacia of the thyroid cartilage.

### 2.2. Methods

Preterm infants were divided into four groups according to gestational age: <29 weeks, 29–32 weeks, 32–34 weeks and 34–37 weeks.

The follow-up process proceeded as follows: Two weeks after discharge, once a month before the corrected age of 6 months and once every two months after the corrected age of 6 months, preterm infants were regularly followed up at the children's health clinic for growth monitoring and physical development evaluation according to the age and growth curve. Feeding advice and guidance were disseminated at these appointments. The follow-up work for preterm infants after discharge referred to the 2019 edition of “Follow-up and Management Recommendations for Preterm Infants After Discharge” (10). Post-discharge nutritional intervention methods were based on the 2016 edition of “Recommendations for Post-Discharge Feeding of Premature and Low Birth Weight Infants” (11) and adopted a personalised feeding program plus family feeding guidance. Some preterm infants were excluded from the study due to NEC or loss to follow-up. The body weight, length, head circumference and milk volume at 40 weeks, 3 months, 6 months and 12 months corrected age, the start oral feeding time, the full oral feeding time, the days of hospitalisation and the catch-up growth were all used as outcome indicators. A parent or guardian signed the informed consent form for each participant.

#### 2.2.1. Interventional group

During hospitalisation in the NICU, preterm infants started training when their vital signs were stable (including when using a non-invasive ventilator). All preterm infants were laid supine in a warm box for training, and a speech trainer who had received professional training and passed an examination completed the intervention. If the preterm infants were receiving auxiliary ventilation, their heart rate and blood oxygen were monitored, and training was immediately stopped in the case of any abnormality.

Oral training refers to the oral exercise intervention program formulated by Fucile et al. (8) and translated by Lu Tianchan et al. (12) The completion of this program takes 15 min, once a day, including 12 min of oral stimulation, which is the stimulation of lips, cheeks, gums and tongue, and 3 min of non-nutritive sucking. Specifically, it comprises perioral stimulation to the cheeks, lips, and jaw for 7 min, intraoral stimulation to the gums and tongue for 5 min, and non-nutritive sucking on a pacifier for 3 min. In this study, the control group used this method of training. In addition to the above oral training method, the intervention group completed breathing training and soft palate lifting function training, a technique collectively known as early combined training.

Breathing training method: The preterm infant is placed in a supine position and retains a symmetrical posture, and the speech trainer stands near the preterm infant's feet and faces the child. (1) The speech trainer places their hands on the left and right rib edges of the preterm infant and pushes their chest slightly from bottom to top and from outside to inside several times; (2) the speech trainer puts one hand on the chest of the preterm infant and massages it clockwise with the palm and fingertips; (3) preterm infants with a lot of phlegm go through body position drainage and expectoration training. Respiratory intervention should be carried out 10 min after oral training for 5–10 min each time, once or twice per day.

Soft palate lifting function training method: The speech trainer presses down the preterm infant's tongue with a tongue depressor to expose the soft palate and quickly applies horizontal stimulation to the soft palate with a frozen cotton swab head to reduce aspiration and choking. This is done once per day, 1–2 s each time.

Our hospital typically uses a deeply hydrolysed formula for feeding, and preterm infants with breast milk will change to breastfeeding after they can be fully fed orally. During the experiment, all the children were fed formula milk.

#### 2.2.2. Observation indicators

The physical index of preterm infants in the intervention group and the control group with different gestational ages were observed at four ages: 40 weeks, 3 months, 6 months and 12 months corrected age. In addition, the catch-up growth satisfaction rate of preterm infants was recorded at these four time points. The preterm postnatal follow-up study (PPFS) of the International Fetal and Newborn Growth Consortium for the 21st Century (INTERGROWTH-21st) provides new growth standards for the evaluation of body weight, length and head circumference (13). We defined the catch-up satisfaction rate as the number of preterm infants whose physical growth index reached the 25th percentile of the growth standards of the PPFS of the INTERGROWTH-21st at corrected months (11) divided by the total number of preterm infants in this group. To directly reflect the catch-up satisfaction rate of physical development in each group, we compared the catch-up satisfaction rate of each physical growth index in different gestational age intervention groups and the catch-up satisfaction rate between the intervention group and the control group for different gestational ages.

### 2.3. Statistical methods

The data in this study were statistically analysed using SPSS 21.0 software. The measurement data were described by $\bar{X}\pm s$, the independent sample T-test was used for comparison between groups and the percentile was used for the description of counting data. The chi-square test and Fisher's exact probability analysis were used for comparison between groups, with *P* < 0.05 indicating a statistical difference.

## 3. Results

### 3.1. Comparison of the general characteristics of preterm infants

The study included a total of 144 preterm infants, 74 males and 70 females. Table 1 shows the results of comparisons between groups of different gestational ages. There was no significant difference in sex, mode of delivery, birth weight, birth length and auxiliary ventilation between the control and intervention groups among the four gestational age groups (*P* > 0.05).

**Table 1:** Comparison of baseline population characteristics of preterm infants.

Project	<29 w		29–32 w		32–34 w		34–37	
	Control	Intervention	Control	Intervention	Control	Intervention	Control	Intervention
Gender male	6	7	9	11	9	10	10	12
(Number) female	5	4	5	6	10	13	13	14
* X*^2^ value	0.188		0.001		0.064		0.035	
* P* value	>0.05		>0.05		>0.05		>0.05	
Natural delivery	4	5	4	4	7	8	14	19
Cesarean section	7	6	10	13	12	15	9	7
* X*^2^ value	0.188		0.102		0.019		0.827	
* P* value	>0.05		>0.05		>0.05		>0.05	
Birth weight (g)	1.13 ± 0.18	1.14 ± 0.19	1.43 ± 0.30	1.50 ± 0.33	1.78 ± 0.27	1.86 ± 0.37	2.33 ± 0.31	2.40 ± 0.45
* T* value	−0.126		−0.556		−0.828		−0.675	
* P* value	>0.05		>0.05		>0.05		>0.05	
Birth length (cm)	37.8 ± 1.96	38.1 ± 2.18	40.2 ± 3.02	40.8 ± 3.03	42.5 ± 3.19	43.1 ± 3.02	46.4 ± 1.73	46.3 ± 2.38
* T* value	−0.278		−0.504		−0.629		0.107	
* P* value	>0.05		>0.05		>0.05		>0.05	
**Auxiliary ventilation**								
* *Yes	9	8	10	12	10	11	7	9
* *No	2	3	4	5	9	12	16	17
* X*^2^ value	0.259		0.003		0.096		0.097	
* P* value	>0.05		>0.05		>0.05		>0.05	

### 3.2. Comparison of the feeding process and hospitalisation days between the intervention group and control group of preterm infants with different gestational ages

The start oral feeding time and the full oral feeding time in the intervention groups of <29 weeks, 29–32 weeks and 32–34 weeks preterm infants were shorter than those in the control groups. The days of hospitalisation in the intervention groups of <29 weeks and 29–32 weeks preterm infants were also shorter than that in the control groups (*P* < 0.05). There was no difference in those three indexes between the intervention group and control group of 34–37 weeks preterm infants (*P* > 0.05) as shown in Table 2.

**Table 2:** Comparison of feeding process and hospitalization days between intervention group and control group of preterm infants with different gestational ages ($\bar{X}\pm s$).

	Control	Intervention	*T* value	*P* value
**<29 w**
Start oral feeding time (days)	17.73 ± 2.01	15.55 ± 1.86*	2.644	0.016
Full oral feeding time (days)	33.64 ± 3.93	30.09 ± 3.86*	2.135	0.045
Days of hospitalization (days)	51.29 ± 9.02	43.00 ± 7.34*	2.360	0.029
**29–32 w**
Start oral feeding time (days)	19.50 ± 2.88	15.18 ± 2.51*	4.473	0.000
Full oral feeding time (days)	27.86 ± 4.13	22.76 ± 3.77*	3.587	0.001
Days of hospitalization (days)	44.64 ± 9.66	35.41 ± 8.54*	2.824	0.008
**32–34 w**
Start oral feeding time (days)	12.89 ± 3.50	10.0 ± 3.16*	2.816	0.008
Full oral feeding time (days)	17.05 ± 3.21	15.0 ± 3.10*	2.102	0.042
Days of hospitalization (days)	28.63 ± 6.09	25.04 ± 6.45	1.840	0.073
**34–37 w**
Start oral feeding time (days)	5.52 ± 2.41	5.54 ± 2.08	−0.026	0.979
Full oral feeding time (days)	7.96 ± 2.72	8.15 ± 2.03	−0.29	0.773
Days of hospitalization (days)	15.22 ± 3.04	15.85 ± 3.75	−0.639	0.526

* Compared with the control group, the intervention group of different gestational age groups has statistical differences.

### 3.3. Comparison of the physical development of preterm infants with different gestational ages at 40 weeks and 3 months corrected age

At 40 weeks and 3 months corrected age, the milk volume, body weight, length and head circumference of preterm infants in the intervention groups were higher than those in the control groups. The difference was statistically significant (*P* < 0.05); see Tables 3, 4 for details.

**Table 3:** Comparison of physical development of preterm infants with different gestational ages at 40 weeks corrected age.

Group		Milk volume (ml/d)	Body weight (g)	Length (cm)	Head circumference (cm)
<29 w	Control	378 ± 20.9	2.63 ± 0.13	47.28 ± 0.69	32.30 ± 0.29
	Intervention	453 ± 58.6*	2.97 ± 0.38*	49.18 ± 1.89*	33.90 ± 1.20*
	*T* value	−3.997	−2.756	−3.314	−4.289
	*P* value	0.002	0.017	0.008	0.001
29-32 w	Control	413 ± 33.3	2.81 ± 0.18	48.07 ± 0.92	32.94 ± 0.49
	Intervention	509 ± 79.2*	3.40 ± 0.52*	51.00 ± 2.51*	35.04 ± 1.46*
	*T* value	−4.503	−4.334	−4.456	−5.545
	*P* value	<0.001	<0.001	<0.001	<0.001
32-34 w	Control	416 ± 24.8	2.77 ± 0.17	48.21 ± 1.22	33.08 ± 0.68
	Intervention	486 ± 72.5*	3.23 ± 0.48*	50.57 ± 2.31*	34.36 ± 1.23*
	*T* value	−4.269	−4.254	−4.236	−4.260
	*P* value	<0.001	<0.001	<0.001	<0.001
34-37 w	Control	471 ± 59.0	2.98 ± 0.29	49.78 ± 1.50	33.71 ± 0.94
	Intervention	514 ± 74.3*	3.47 ± 0.61*	51.18 ± 2.39*	34.76 ± 1.60*
	*T* value	−2.248	−3.710	−2.498	−2.845
	*P* value	0.029	0.001	0.016	0.007

* There were significant differences between the intervention group and the control group with different gestational age groups.

**Table 4:** Comparison of physical development of preterm infants with different gestational ages at 3 months corrected age.

Group		Milk volume (ml/d)	Body weight (g)	Length (cm)	Head circumference (cm)
<29 w	Control	862 ± 66.9	5.59 ± 0.37	58.26 ± 1.53	38.76 ± 0.74
	Intervention	985 ± 137.6*	6.46 ± 1.18*	62.00 ± 3.61*	40.37 ± 1.6*
	*T* value	−2.660	−2.534	−3.159	−3.005
	*P* value	0.015	0.026	0.005	0.0071
29-32 w	Control	893 ± 36.4	5.96 ± 0.24	60.44 ± 1.11	39.50 ± 0.78
	Intervention	1028 ± 159.1*	6.85 ± 1.07*	63.45 ± 2.83*	40.61 ± 1.65*
	*T* value	−3.387	−3.358	−4.011	−2.454
	*P* value	0.003	0.004	0.001	0.022
32-34 w	Control	895 ± 55.0	5.96 ± 0.37	60.77 ± 1.08	39.52 ± 0.64
	Intervention	1035 ± 129.9*	6.90 ± 0.87*	63.66 ± 3.12*	40.29 ± 1.10*
	*T* value	−4.694	−4.719	−4.141	−2.819
	*P* value	<0.001	<0.001	<0.001	0.008
34-37 w	Control	954 ± 92.3	6.36 ± 0.62	61.63 ± 1.56	39.72 ± 1.19
	Intervention	1060 ± 162.0*	7.07 ± 1.08*	63.71 ± 2.18*	40.90 ± 1.20*
	*T* value	−2.856	−2.851	−3.855	−3.459
	*P* value	0.007	0.007	<0.001	0.001

* There were significant differences between the intervention group and the control group with different gestational age groups.

### 3.4. Comparison of the physical development of preterm infants with different gestational ages at 6 months corrected age

As shown in Table 5, the milk volume, body weight, length and head circumference of preterm infants in the <29 week intervention group were still higher than those in the control group (*P* < 0.05); the body weight and length of the 29–32 and 32–34 weeks intervention groups were higher than those in the control group (*P* < 0.05). The body weight and head circumference of the 34–37 weeks intervention group were higher than those of the control group (*P* < 0.05).

**Table 5:** Comparison of physical development of preterm infants with different gestational ages at 6 months corrected age.

Group		Milk volume (ml/d)	Body weight (g)	Length (cm)	Head circumference (cm)
<29 w	Control	1061 ± 46.0	7.08 ± 0.32	65.00 ± 1.31	41.99 ± 0.70
	Intervention	1207 ± 158.2*	8.05 ± 1.05*	68.73 ± 2.86*	42.85 ± 1.13*
	*T* value	−2.939	−2.898	−3.933	−2.129
	*P* value	0.013	0.014	0.001	0.046
29-32w	Control	1065 ± 113.6	7.59 ± 0.28	67.64 ± 0.90	43.17 ± 0.87
	Intervention	1160 ± 116.7*	8.95 ± 1.49*	69.56 ± 3.23*	43.69 ± 1.61
	*T* value	−2.279	−3.672	−2.343	−1.088
	*P* value	0.03	0.002	0.030	0.286
32-34 w	Control	1053 ± 130.2	7.47 ± 0.35	67.29 ± 0.95	42.51 ± 0.79
	Intervention	1145 ± 163.7	8.31 ± 1.07*	69.55 ± 3.40*	43.08 ± 1.39
	*T* value	−2.002	−3.532	−3.044	−1.689
	*P* value	0.052	0.001	0.005	0.100
34-37 w	Control	1050 ± 150.4	7.80 ± 0.63	68.27 ± 2.84	42.57 ± 1.43
	Intervention	1041 ± 189.1	8.72 ± 0.10*	69.77 ± 2.68	43.67 ± 1.21*
	*T* value	0.184	−3.752	−1.896	−2.889
	*P* value	0.855	<0.001	0.064	0.006

* There were significant differences between the intervention group and the control group with different gestational age groups.

### 3.5. Comparison of the physical development of preterm infants with different gestational ages at a 12-month corrected age

At the corrected age of 12 months, the body weight and length of preterm infants in the <29 weeks intervention group were still higher than those in the control group. The body weight of preterm infants in the 29–32 and 32–34 weeks intervention groups was higher than in the control groups (*P* < 0.05). There was no statistical difference between the two groups in the 34–37 weeks group (*P* > 0.05) as shown in Table 6.

**Table 6:** Comparison of physical development of preterm infants with different gestational ages at 12 months corrected age.

Group		Milk volume (ml/d)	Body weight (g)	Length (cm)	Head circumference (cm)
<29 w	Control	450 ± 176.1	8.91 ± 0.55	74.63 ± 1.94	45.05 ± 0.59
	Intervention	600 ± 180.3	9.83 ± 1.29*	76.86 ± 2.85*	45.64 ± 1.44
	*T* value	−1.974	−2.174	−2.155	−1.239
	*P* value	0.062	0.048	0.044	0.237
29-32 w	Control	491 ± 136.5	9.31 ± 0.52	76.22 ± 0.87	45.26 ± 1.03
	Intervention	515 ± 136.8	10.13 ± 1.38*	77.21 ± 3.10	46.08 ± 1.57
	*T* value	−0.498	−2.267	−1.249	−1.661
	*P* value	0.622	0.034	0.227	0.108
32-34 w	Control	606 ± 203.3	9.36 ± 0.47	76.77 ± 1.14	45.69 ± 0.76
	Intervention	549 ± 201.3	10.07 ± 1.03*	77.52 ± 3.09	45.77 ± 1.52
	*T* value	0.915	−2.962	−1.076	−0.207
	*P* value	0.366	0.006	0.291	0.837
34-37 w	Control	679 ± 211.1	9.74 ± 0.74	77.47 ± 1.51	45.71 ± 1.06
	Intervention	650 ± 249.1	10.21 ± 0.99	78.54 ± 2.48	46.27 ± 1.20
	*T* value	0.444	−1.851	−1.852	−1.723
	*P* value	0.659	0.070	0.071	0.091

* There were significant differences between the intervention group and the control group with different gestational age groups.

### 3.6. Comparison of the physical growth catch-up satisfaction rate of preterm infants in different gestational age groups at 40 weeks

At 40 weeks of corrected age, there was no significant difference in the catch-up satisfaction rate of body weight, length and head circumference between the <29, 29–32, 32–34 and 34–37 weeks intervention groups. However, the catch-up satisfaction rate in the intervention group was higher than in the control group at all gestational ages (*P* < 0.05), except for the weight of the <29 weeks group and the weight and length of the 34–37 weeks group; in these three cases, there was no significant difference as shown in Table 7.

**Table 7:** Comparison of the satisfaction rate of physical development catch-up in different gestational age groups (%) group.

		40 weeks CA.			3 months CA.			6 months CA.			12 months CA.		
		W	L	H	W	L	H	W	L	H	W	L	H
<29 w	CG.	9.10	9.10	0	9.10	9.10	9.10	9.10	9.10	9.10	36.40	18.20	9.10
	IG.	36.40	54.50*	54.50*	54.50*	81.80*	54.50*	63.60*	81.80*	27.30	81.80	72.70*	45.50
	*X* ^2^	2.330	5.240	7.880	5.240	11.730	5.240	7.070	11.730	1.220	2.930	4.700	3.670
	*P*	0.130	0.020	0.012	0.020	0.001	0.020	0.008	0.001	0.269	0.087	0.030	0.056
29-32 w	CG.	7.10	7.10	14.30	7.10	21.40	28.60	14.30	50.00	64.30	42.90	64.30	42.90
	IG.	76.50*	70.60*	76.50*	94.10*^#^	100.00*	64.70*	94.10*	94.10*	76.50^#^	94.10*^#^	100.00*^#^	70.60^#^
	*X* ^2^	17.390	12.690	11.890	23.450	20.000	4.010	23.000	7.810	0.550	9.790	7.000	2.430
	*P*	<0.001	<0.001	0.001	<0.001	<0.001	0.040	<0.001	0.005	0.457	0.002	0.008	0.12
32-34 w	CG.	0	26.30	26.30	5.30	26.30	36.80	31.60	52.60	42.10	63.20	73.70	73.70
	IG.	56.50*	82.60*	56.50*	95.70*^#^	100.00*	78.30*^#^	95.70*^#^	95.70*	65.20	95.70*^#^	100.00*^#^	65.20^#^
	*X* ^2^	15.180	13.460	3.876	37.220	21.790	7.409	19.220	13.540	2.244	9.930	6.710	0.349
	*P*	<0.001	<0.001	0.049	<0.001	<0.001	0.006	<0.001	<0.001	0.134	0.002	0.010	0.555
34-37 w	CG.	52.20	78.30	52.20	56.50	56.50	56.50	60.90	87.00	56.50	91.30	91.30	78.30
	IG.	76.90	88.50	80.80*	92.30*^#^	92.30*	96.20*^#^	100.00*^#^	96.20	92.30*^#^	100.00^#^	100.00^#^	92.30^#^
	*X* ^2^	3.299	0.930	4.538	8.452	8.452	11.010	12.210	3.540	11.010	2.310	2.310	1.970
	*P*	0.069	0.335	0.033	0.004	0.004	0.001	<0.001	0.060	0.001	0.129	0.129	0.161
Gestational age group	*X* ^2^	2.274	5.713	4.812	12.36	3.355	10.867	11.339	7.315	19.240	8.596	12.880	10.218
	*P*	0.132	0.110	0.185	0.001	0.319	0.009	0.001	0.063	<0.001	0.004	<0.001	0.014

* There were significant differences between the intervention group and the control group with different gestational age groups.

# There were significant differences between the different gestational intervention groups and the <29w's intervention group.

CA, corrected age; W, body weight; L, length; H,head circumference; CG, control group; IG, intervention group; *P*, *P* value.

### 3.7. Comparison of the physical growth catch-up satisfaction rate of preterm infants in different gestational age groups at 3 months

At 3 months of corrected age, there is no statistical difference in the body length catch-up satisfaction rate between the four gestational groups (*P* > 0.05). However, the catch-up growth of body weight and head circumference is different between the four gestational groups. Except for the index of the 29–32 weeks head circumference, the body weight and head circumference catch-up satisfaction rates of preterm infants in the intervention groups of >29 weeks were higher than those of <29 weeks (*P* < 0.05). Among the four gestational age groups, the catch-up satisfaction rates of body weight, body length and head circumference in the intervention group were higher than those in the control group, and the differences were statistically significant (*P* < 0.05) as shown in Table 7.

### 3.8. Comparison of the physical growth catch-up satisfaction rate of preterm infants in different gestational age groups at 6 months

At the corrected age of 6 months, there was no difference in the body length catch-up satisfaction rate among the four gestational age intervention groups. The catch-up satisfaction rate of the head circumference of the 29–32 weeks intervention group, the body weight of the 32–34 weeks intervention group and the body weight and head circumference of the 34–37 weeks intervention group were all higher than those of the <29 weeks intervention group (*P* < 0.05). The body weight and length catch-up satisfaction rates of the three <34 weeks intervention groups were higher than those of the control groups (*P* < 0.05), as were the body weight and head circumference of the 34–37 weeks intervention group (*P* < 0.05), but there was no significant difference in the length catch-up satisfaction rate between the 34–37 weeks intervention group and its control group as shown in Table 7.

### 3.9. Comparison of the physical growth catch-up satisfaction rate of preterm infants in different gestational age groups at 12 months

At the corrected age of 12 months, the catch-up satisfaction rates of body weight, length and head circumference in the three >29 weeks intervention groups were higher than those in the <29 weeks intervention group (*P* < 0.05). The length catch-up satisfaction rate in the <29 weeks intervention group was higher than that in the control group (*P* < 0.05), and there was no difference in body weight and head circumference catch-up satisfaction rate between the two groups. The body weight and length catch-up satisfaction rates of the 29–32 weeks and 32–34 weeks intervention groups were higher than those of the control groups (*P* < 0.05), and there was no difference in the head circumference catch-up satisfaction rate between the two groups. The same is true of the body weight, length and head circumference of the 34–37 weeks group (*P* > 0.05) as shown in Table 7.

## 4. Discussion

Swallowing function training can promote the development of the feeding function of preterm infants, increase sucking strength and milk volume, shorten the time to achieve full oral feeding and thus accelerate the development of body weight (14). There is significant existing literature (15, 16) on swallowing training to promote oral feeding of preterm infants, based on oral exercise training. We added breathing training and soft palate lifting function training to construct a systematic intervention program, which we call early combined training. According to the preterm infant's breathing characteristics, a passive breathing exercise is carried out, with the intervener stimulating the abdomen to induce breathing (17). By lifting the palms and pressing down according to the rhythm of the newborn's exhalation and inhalation, one can effectively regulate the respiratory rhythm of preterm infants, assist in improving their breathing strength, and promote the coordination ability of the preterm infant's breathing and swallowing, all of which improves their oral feeding ability (18, 19). This increases the pressure in the oral cavity and the power of food moving from the oral cavity through the pharynx to the oesophageal entrance by training the soft palate lifting function.

Preterm infants' nutritional intake not only has to meet their own basal metabolism but also requires stimulating catch-up growth to achieve the growth rate of normal newborns (20, 21). Body weight is the most direct essential indicator of the growth and development of preterm infants. Research and observation indicators differ when it comes to body weight gain. In the past, more attention was paid to the influence of oral training on the body weight of preterm infants (i.e., fully orally fed and discharged from the hospital), and more short-term effects were observed (15, 16). Our study not only observed the physical development of preterm infants with different gestational ages from the corrected age of 40 weeks to 12 months but also observed the catch-up growth within the first year of birth. The results show that, in the <29 weeks group, the physical development of preterm infants receiving early combined training was higher than in the simple oral training group at four observation time points. More than half of the preterm infants (54.5%) at the corrected age of 40 weeks had reached the catch-up satisfaction growth of length and head circumference. The catch-up satisfaction rate of physical development gradually increased at the corrected ages of 3 months, 6 months and 12 months, and the catch-up satisfaction rates of body weight, length and head circumference at 12 months corrected age reached 81.8%, 72.7% and 45.5%, respectively, suggesting that early combined training could promote the physical development of <29 weeks preterm infants; the body weight will catch up first, then the length and finally the head circumference. This is consistent with results obtained by Gao Xiaoyan, who conducted regular feeding instructions and follow-up monitoring of preterm infants after discharge to analyse and correct growth and development within six months (22).

The physical development indexes of preterm infants in the 29–32 weeks and 32–34 weeks groups receiving early combined training were higher than those of the control group at 40 weeks and 3 months corrected age. However, there was no difference in head circumference between the intervention group and the control group at 6 months and 12 months of corrected age. Several studies have concurred that the physical growth speed of preterm infants is the fastest within the first three months, is relatively slower between three and six months and slows down even more after six months, presenting a growth trend of “first, fast and then slow” (23, 24). The length catch-up satisfaction rate of preterm infants in these two gestational age groups reached 100% at 3 months of corrected age, and the body weight and head circumference catch-up satisfaction rates of the 29–32 weeks group were 94.1% and 64.7%, respectively, while in the 32–34 weeks group these were 95.7% and 78.3%, respectively, suggesting that early combined training could promote the early physical development of 29–34 weeks preterm infants. For this gestational age, the length catches up first, then the body weight and finally the head circumference.

The 34–37 weeks preterm infants who received early combined training had higher physical development than the control group before the corrected age of 3 months, but there was no difference in body length between the intervention group and the control group at 6 months of corrected age, and neither of the physical development indexes at 12 months corrected age. The body weight catch-up satisfaction rate of the 34–37 weeks intervention group was 100% at the corrected age of 6 months, and it remained 100% at 12 months corrected age along with 100% for length and 92.3% for head circumference. Compared with the control group, there was no difference, suggesting that early combined training can promote the early physical development of 34–37 weeks preterm infants, but it has no significant effect on long-term physical growth. For 34–37 weeks preterm infants, body weight catches up first, then the length and finally the head circumference.

The body weight reflects the short-term nutritional status, while the length reflects the long-term nutritional status. In this study, the body weight catch-up satisfaction rate of <29 weeks and 34–37 weeks preterm infants occurred first, followed by the length and finally the head circumference, consistent with previous research results (25). The length catch-up satisfaction rate of 29–34 weeks preterm infants occurred first, then the body weight and finally the head circumference. These results were different from previous research. A possible reason for this is good in-hospital nutrition and infection or other problems following discharge affecting the catch-up rates.

This study has some limitations. While it focused on the effect of early combined training on the physical development of preterm infants of different gestational ages, it did not address neuropsychological or mental development. Neurobehavioral development has a significant impact on newborn growth as it helps stabilise all subsystems, provides a means to exhibit signs of stress and strengths and plays a fundamental role in detecting the effects of interventions on this category of newborns. Therefore, it is necessary to comprehensively explore the effects of early combined training on the physical and psychological development of preterm infants in future studies. Additionally, due to selection limitations, the included samples may have been biased, and more large-sample, multicentre follow-up studies may be needed in the future to further investigate the balanced catch-up growth concerning weight, length and head circumference in preterm infants.

## 5. Conclusion

In summary, early combined training can promote early catch-up growth in preterm infants, particularly in early gestational age groups (<34 weeks). The older the gestational age, the sooner catch-up growth satisfaction will occur. However, there is an imbalance in the chase speed of weight, length and head circumference. Early hospitalisation, combined training, regular follow-up after discharge and standardised nutrition management can significantly promote the physical development of preterm infants.

## Data Availability

The original contributions presented in the study are included in the article/Supplementary Material, further inquiries can be directed to the corresponding author/s.
